# Knowledge Mapping and Global Trends in Simulation in Medical Education: Bibliometric and Visual Analysis

**DOI:** 10.2196/71844

**Published:** 2025-03-26

**Authors:** Hongjun Ba, Lili Zhang, Xiufang He, Shujuan Li

**Affiliations:** 1 Department of Pediatrics The First Affiliated Hospital Sun Yat-sen University Guangzhou China; 2 Department of Pediatric Cardiology The First Afﬁliated Hospital Sun Yat-sen University Guangzhou China

**Keywords:** medical education, simulation-based teaching, bibliometrics, visualization analysis, knowledge mapping

## Abstract

**Background:**

With the increasing recognition of the importance of simulation-based teaching in medical education, research in this field has developed rapidly. To comprehensively understand the research dynamics and trends in this area, we conducted an analysis of knowledge mapping and global trends.

**Objective:**

This study aims to reveal the research hotspots and development trends in the field of simulation-based teaching in medical education from 2004 to 2024 through bibliometric and visualization analyses.

**Methods:**

Using CiteSpace and VOSviewer, we conducted bibliometric and visualization analyses of 6743 articles related to simulation-based teaching in medical education, published in core journals from 2004 to 2024. The analysis included publication trends, contributions by countries and institutions, author contributions, keyword co-occurrence and clustering, and keyword bursts.

**Results:**

From 2004 to 2008, the number of articles published annually did not exceed 100. However, starting from 2009, the number increased year by year, reaching a peak of 850 articles in 2024, indicating rapid development in this research field. The United States, Canada, the United Kingdom, Australia, and China published the most articles. Harvard University emerged as a research hub with 1799 collaborative links, although the overall collaboration density was low. Among the 6743 core journal articles, a total of 858 authors were involved, with Lars Konge and Adam Dubrowski being the most prolific. However, collaboration density was low, and the collaboration network was relatively dispersed. A total of 812 common keywords were identified, forming 4189 links. The keywords “medical education,” “education,” and “simulation” had the highest frequency of occurrence. Cluster analysis indicated that “cardiopulmonary resuscitation” and “surgical education” were major research hotspots. From 2004 to 2024, a total of 20 burst keywords were identified, among which “patient simulation,” “randomized controlled trial,” “clinical competence,” and “deliberate practice” had high burst strength. In recent years, “application of simulation in medical education,” “3D printing,” “augmented reality,” and “simulation training” have become research frontiers.

**Conclusions:**

Research on the application of simulation-based teaching in medical education has become a hotspot, with expanding research areas and hotspots. Future research should strengthen interinstitutional collaboration and focus on the application of emerging technologies in simulation-based teaching.

## Introduction

In the rapidly evolving landscape of medical education, the integration of simulation-based training has emerged as a pivotal innovation. Simulation in medical education encompasses a broad spectrum of methodologies, including high-fidelity mannequins, virtual reality, standardized patients, and computer-based simulations [1,2]. These techniques aim to enhance clinical skills, decision-making, and teamwork among medical professionals without the direct involvement of real patients.

The adoption of simulation in medical training addresses several critical challenges [3,4]. First, it provides a safe and controlled environment where learners can practice and refine their skills. This is particularly crucial in high-stakes scenarios such as emergency medicine, surgery, and critical care, where errors can have severe consequences [5,6]. In addition, simulation allows for repetitive practice and immediate feedback, facilitating a deeper understanding of complex procedures and concepts.

Over the past few decades, there has been a significant increase in research focused on the effectiveness and impact of simulation-based education in the medical field [7,8]. This growing body of literature reflects the widespread recognition of simulation as a valuable educational tool. However, the rapid expansion of this field necessitates a comprehensive review and analysis to understand its development, trends, and future directions.

Several bibliometric analyses have been conducted on simulation in medical education [9,10], highlighting its growing importance and impact. However, these studies often focus on specific aspects of simulation, such as surgical training or virtual reality. Our study complements this body of research by providing a comprehensive overview of the entire field, including emerging technologies like 3D printing and augmented reality (AR), and by analyzing collaborative networks and thematic trends over a 20-year period.

A bibliometric analysis provides an ideal approach to systematically evaluate the literature on simulation in medical education. By using quantitative methods to analyze publication patterns, citation networks, and research themes, bibliometric studies can offer valuable insights into the evolution of this field. Such an analysis can identify key contributors, influential publications, and emerging trends, thereby guiding future research and practice.

This study aims to conduct a bibliometric analysis of the literature on simulation in medical education. By examining the scope, growth, and impact of research in this area, we seek to elucidate the current state of the field and identify potential gaps and opportunities for further investigation. Specifically, this analysis will focus on the following objectives:

1. To map the overall publication trends and growth in simulation-based medical education research.
2. To identify the most influential journals, articles, and authors contributing to this field.
3. To explore the thematic evolution and emerging trends within the literature.
4. To assess the collaborative networks and geographical distribution of research activities.

Through this comprehensive bibliometric analysis, we hope to provide a clearer understanding of the trajectory and impact of simulation in medical education, ultimately contributing to the enhancement of educational practices and outcomes in the medical field.

## Methods

### Data Acquisition and Search Strategy

The search was conducted in the Web of Science Core Collection (WoSCC) database, which is widely recognized for its comprehensive coverage of high-quality, peer-reviewed literature [11,12]. While we acknowledge that including additional databases such as PubMed or Scopus could provide a more comprehensive dataset, the WoSCC was chosen for its superior bibliographic accuracy and extensive coverage of medical education research. Therefore, we opted to perform our search within this database. We conducted a search in the Web of Science for all relevant papers published between January 1, 2004, and December 31, 2024. The time frame from January 1, 2004, to December 31, 2024, was selected because it marks the period when simulation-based medical education began to gain significant traction in the literature, reflecting the growing recognition of its importance in medical training. In medical education, we define “simulation” as a teaching and training method that encompasses high-fidelity mannequins, virtual reality, standardized patients, and computer-based simulations.

The search formula “TS=(Medical education) AND TS=(Simulation)” was used. The inclusion criteria were as follows: (1) full-text publications related to simulation in medical education, including original research articles and review articles; (2) articles written in English; and (3) papers published between January 1, 2004, and December 31, 2024. We excluded conference abstracts, theses, dissertations, and nonpeer-reviewed articles to ensure the quality and relevance of the data. The exclusion criteria were (1) topics not related to simulation in medical education and (2) papers in the form of conference abstracts, theses, dissertations, and non–peer-reviewed articles to ensure the quality and relevance of the data. A plain text version of the papers was exported.

### General Data

**Figure 1:**
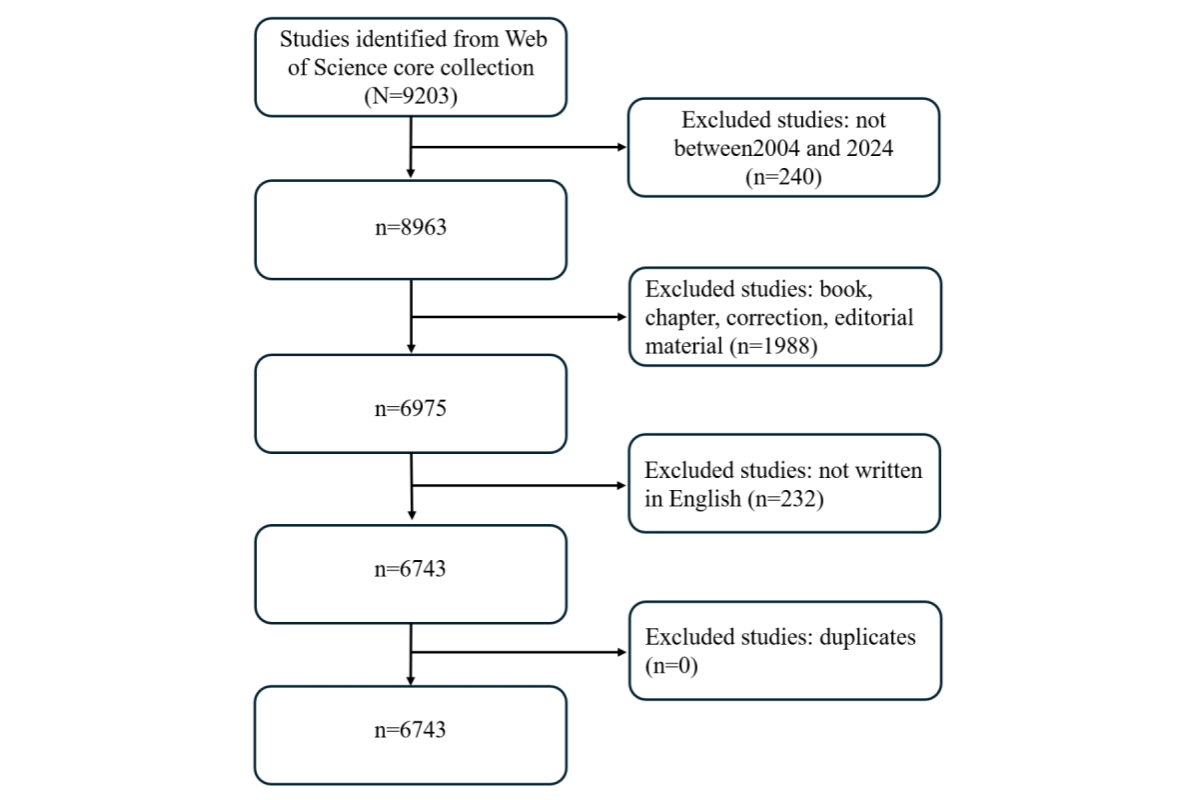
The workflow of data collection and bibliometric analysis.

Between January 1, 2004, and December 31, 2024, the WoSCC database recorded a total of 6743 publications concerning simulation in medical education. This body of literature included contributions from 121 countries and regions, 510 institutions, and 858 authors. Figure 1 shows the process of literature searching and bibliometric analysis.

### Data Analysis

We used GraphPad Prism (version 8.0.2; Dotmatics) to illustrate annual publication trends. The methodological approach was validated through the use of CiteSpace and VOSviewer, both of which are widely recognized and extensively used in bibliometric research [13,14]. These tools have been shown to provide robust and reliable analyses of large-scale bibliometric data.

VOSviewer, a Java-based software developed by van Eck and Waltman in 2009, facilitates the construction of various types of network maps, such as bibliographic coupling, cocitation, and coauthorship networks. CiteSpace, developed by Professor Chaomei Chen, provides a dynamic platform for identifying and visualizing patterns and trends in scientific literature, enabling the exploration of knowledge domains and predictive analysis of research trajectories [14]. Our methodological approach involved setting specific parameters for network density (eg, keyword co-occurrence density of 0.0127), node inclusion thresholds (eg, minimum occurrence frequency of keywords), and time-slicing techniques to analyze temporal changes. The references corresponding to the software applications were verified against our citation list to ensure accuracy [13,14]. When using VOSviewer and CiteSpace for bibliometric analysis, we established standards for defining international collaboration. This was done by examining the authorship of papers, specifically the first and corresponding authors, to ensure a comprehensive capture of collaborative efforts from researchers across different countries.

Burst detection in CiteSpace is based on the Kleinberg algorithm, which models document streams using infinite-state automata to extract meaningful structures [15]. These analyses can reveal rapidly growing topics over extended periods as well as short-term themes.

The rationale for selecting these techniques lies in their widespread application and effectiveness in bibliometric research. They provide robust and complementary insights into productivity, impact, and collaboration patterns within research fields.

## Results

### Publication Trend

**Figure 2:**
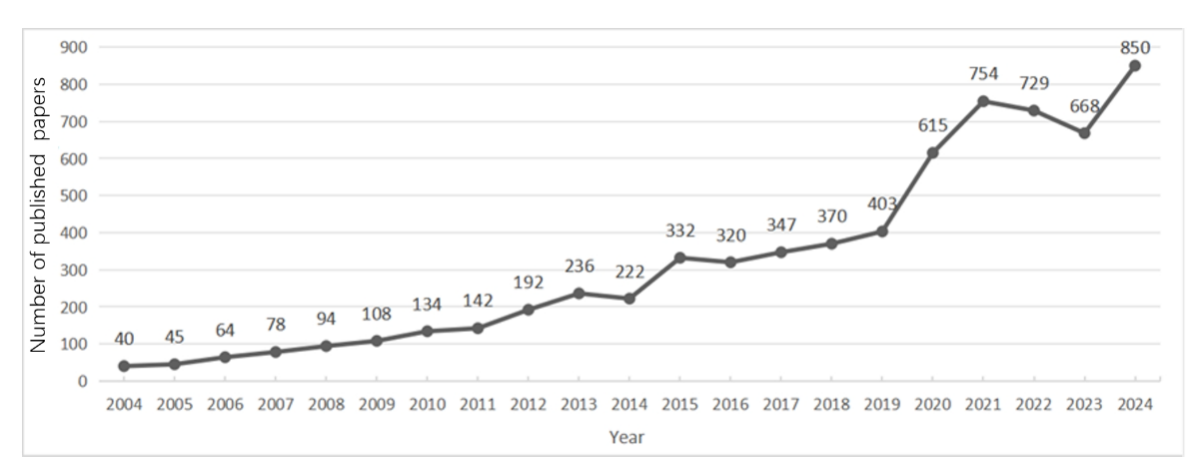
Trend chart of publications in the past 20 years.

Figure 2 shows that from 2004 to 2008, the annual number of publications on simulation teaching in medical education did not exceed 100 articles, indicating that research in this field was still in its nascent stage. Since 2009, the number of publications in this field has steadily increased, showing a trend of fluctuating growth. Specifically, the number of publications in 2015 surpassed 300 for the first time, and by 2020, this number had exceeded 500. This significant increase marks the growing attention and interest of scholars and researchers in the field of simulation teaching in medical education. Since 2020, the annual number of publications in this field has consistently remained above 500, reaching a peak of 850 articles in 2024. This further highlights the vigorous development and extensive influence of research in the field of simulation teaching in medical education.

### Country or Region and Institution Contributions

**Figure 3:**
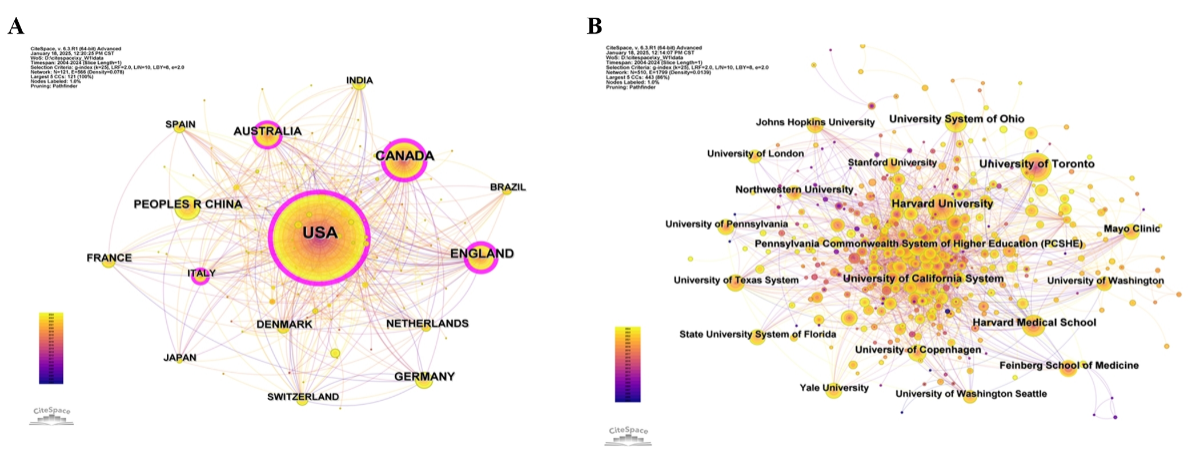
Network graph of national and institutional collaborations. (A) Network graph of national collaborations. (B) Network graph of institutional collaborations. The bubble size represents the number of publications.

According to Figure 3A, the connections between circular nodes representing different countries to some extent reflect the existence of relationships and collaborations between these countries. Furthermore, the density of these connections in the network can serve as an important indicator of the closeness of collaborative relationships between countries. Among them, the countries with the highest number of publications are the United States (3083 articles), Canada (776 articles), England (510 articles), Australia (381 articles), and China (375 articles) (Table 1). In addition, countries such as Italy, the Netherlands, Sweden, and Belgium have numerous connections, indicating a complex network of relationships, which suggests that these countries have relatively close research collaborations with other regions.

Using the CiteSpace software, an institutional collaboration network diagram was obtained, as shown in Figure 3B. Upon statistical analysis, it was found that there are a total of 510 research institutions forming 1799 connections, with Harvard University being the central hub. The diagram reveals that the network density is 0.0139, indicating relatively weak collaborative relationships between research institutions, with a significant portion of them operating in a relatively independent research state. In terms of research output, the top-10 institutions by the number of publications are Harvard University, the University of Toronto, the University of California System, the University System of Ohio, Harvard Medical School, Mayo Clinic, Northwestern University, Feinberg School of Medicine, the University of Copenhagen, and Pennsylvania Commonwealth System of Higher Education (Table 2).

**Table 1:** Top-10 most productive countries or regions.

Rank	Country or region	Articles, n	Centrality	Percentage	Half-life
1	United States	3083	0.51	45.71%	14.5
2	Canada	776	0.22	11.52%	13.5
3	England	510	0.25	7.57%	15.5
4	Australia	381	0.12	5.64%	15.5
5	China	375	0.02	5.56%	15.5
6	Germany	340	0.05	5.04%	16.5
7	France	189	0.03	2.8%	15.5
8	Denmark	184	0.05	2.73%	15.5
9	The Netherlands	164	0.1	2.43%	15.5
10	Switzerland	142	0.05	2.1%	16.5

**Table 2:** Top-10 most productive institutions.

Rank	Institution	Country	Studies, n	Centrality	Half-life
1	Harvard University	United States	167	0.06	13.5
2	University of Toronto	Canada	153	0.04	11.5
3	University of California System	United States	125	0.01	12.5
4	University System of Ohio	United States	118	0.04	11.5
5	Harvard Medical School	United States	82	0.09	13.5
6	Mayo Clinic	United States	68	0.03	12.5
7	Northwestern University	United States	66	0.01	10.5
8	Feinberg School of Medicine	United States	65	0.01	10.5
9	University of Copenhagen	Denmark	61	0.01	14.5
10	Pennsylvania Commonwealth System of Higher Education	United States	60	0.02	13.5

### Author Collaborations

**Figure 4:**
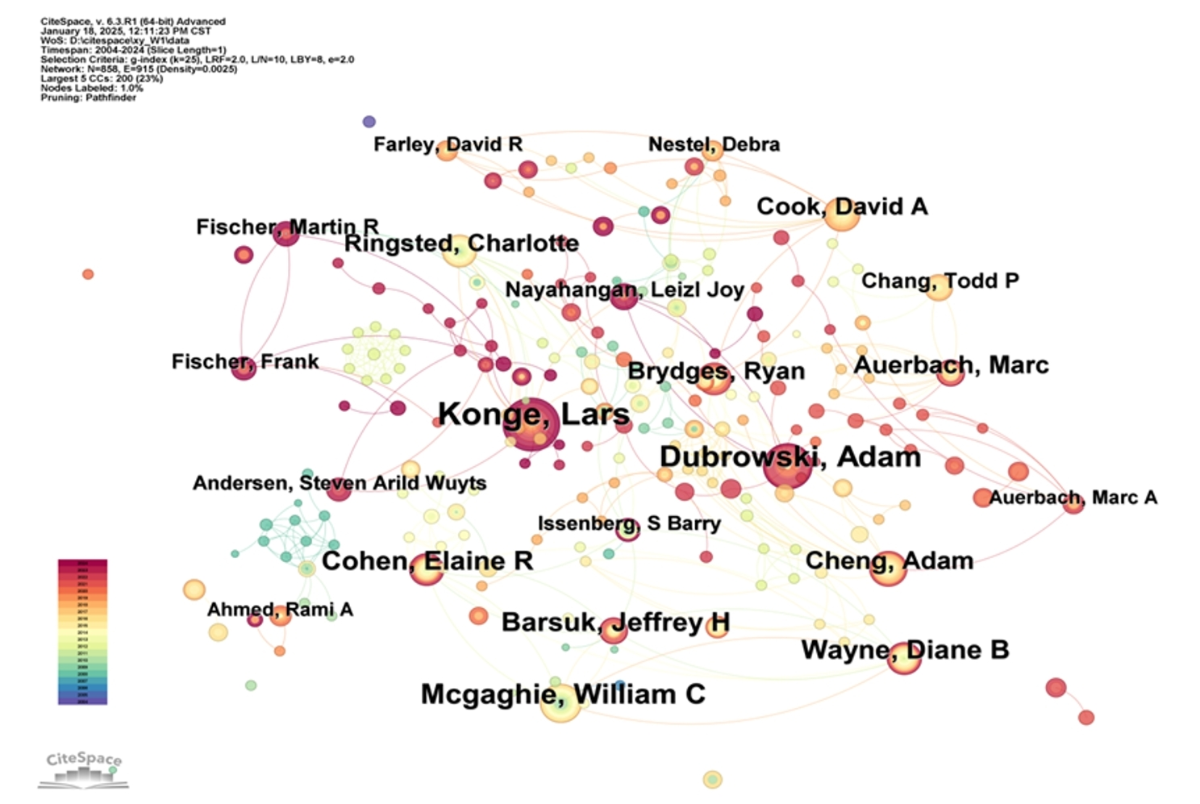
Network diagram of author collaborations. The bubble size represents the number of publications.

The sample data were processed using CiteSpace, and the resulting author co-occurrence map is shown in Figure 4. In this map, each node represents a different author. The size of the node indicates the author’s publication frequency, meaning the larger the node, the more publications the author has. When nodes are presented in the form of annual rings, the bandwidth of the color band corresponding to a particular year represents the number of papers published by the author that year, with a wider ring indicating more publications. The lines between nodes represent collaborative relationships between organizations or authors, with the thickness of the lines indicating the degree of collaboration.

Among the 6743 core journal articles, a total of 858 authors were involved. The top-10 authors by publication volume are Konge, Lars (69 papers); Dubrowski, Adam (50 papers); McGaghie, William C (37 papers); Wayne, Diane B (33 papers); Cohen, Elaine R (33 papers); Barsuk, Jeffrey H (30 papers); Auerbach, Marc (26 papers); Cheng, Adam (24 papers); Cook, David A (23 papers); and Ringsted, Charlotte (22 papers). Authors with 7 or more publications, a total of 44 individuals, were classified as the core author group, which accounts for only 5.1% of the total authors. In addition, there are 915 collaboration lines among the authors on the map, with a collaboration density of 0.0025, indicating a low-density level. The number of lines is relatively sparse, and the collaboration network map shows a relatively dispersed pattern. The largest collaboration network system is formed by the research team centered around Dubrowski, Adam; Nayahangan, Leizl Joy; Cheng, Adam; Auerbach, Marc A; and Cook, David A. The scale of collaboration is mainly presented in the form of individual or small-scale research teams, indicating that the core research team in this field has yet to be fully established.

### Keyword Co-Occurrence and Cluster

**Figure 5:**
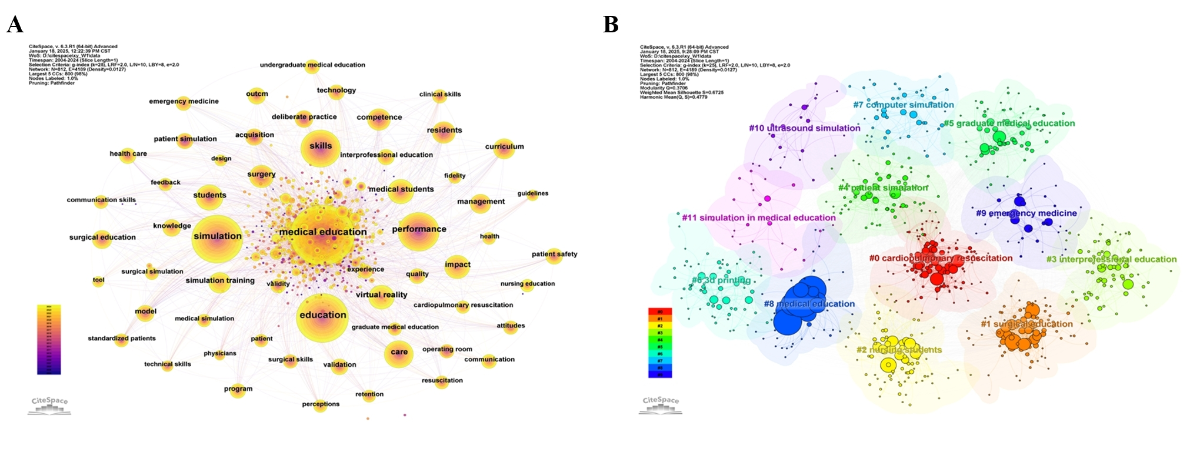
Keyword co-occurrence and keyword clustering map. (A) Keyword co-occurrence map. (B) Keyword clustering map. The bubble size represents the number of publications.

Using CiteSpace software to conduct a keyword co-occurrence analysis on the sample, the constructed keyword co-occurrence map is shown in Figure 5A. From the keyword co-occurrence analysis, a total of 812 common keywords were identified, forming 4189 connections, with a network density of 0.0127. The most frequently occurring keyword is “medical education,” accounting for 7.9%. This is followed by “education” and “simulation,” which account for 5.48% and 5.14%, respectively. The keywords “performance” and “skills” account for 3.49% and 3.29%, respectively. These keywords represent the current research hotspots and status in the field of simulation teaching in medical education.

Based on the keyword co-occurrence map, the log-likelihood ratio algorithm was used to cluster the keywords, resulting in a keyword clustering co-occurrence map. The Q value is 0.3707 (>0.3), and the S value is 0.6725 (>0.5), indicating a significant clustering structure and a high degree of clustering match. The map displays a total of 10 clustering areas, among which “cardiopulmonary resuscitation,” “surgical education,” “nursing students,” “interprofessional education,” and “patient simulation” are the five largest clusters (Figure 5B). Specifically, medical simulation teaching has become an important component of medical education, widely applied in various fields including cardiopulmonary resuscitation, surgical education, and nursing student training.

### Keyword Citation Bursts

**Figure 6:**
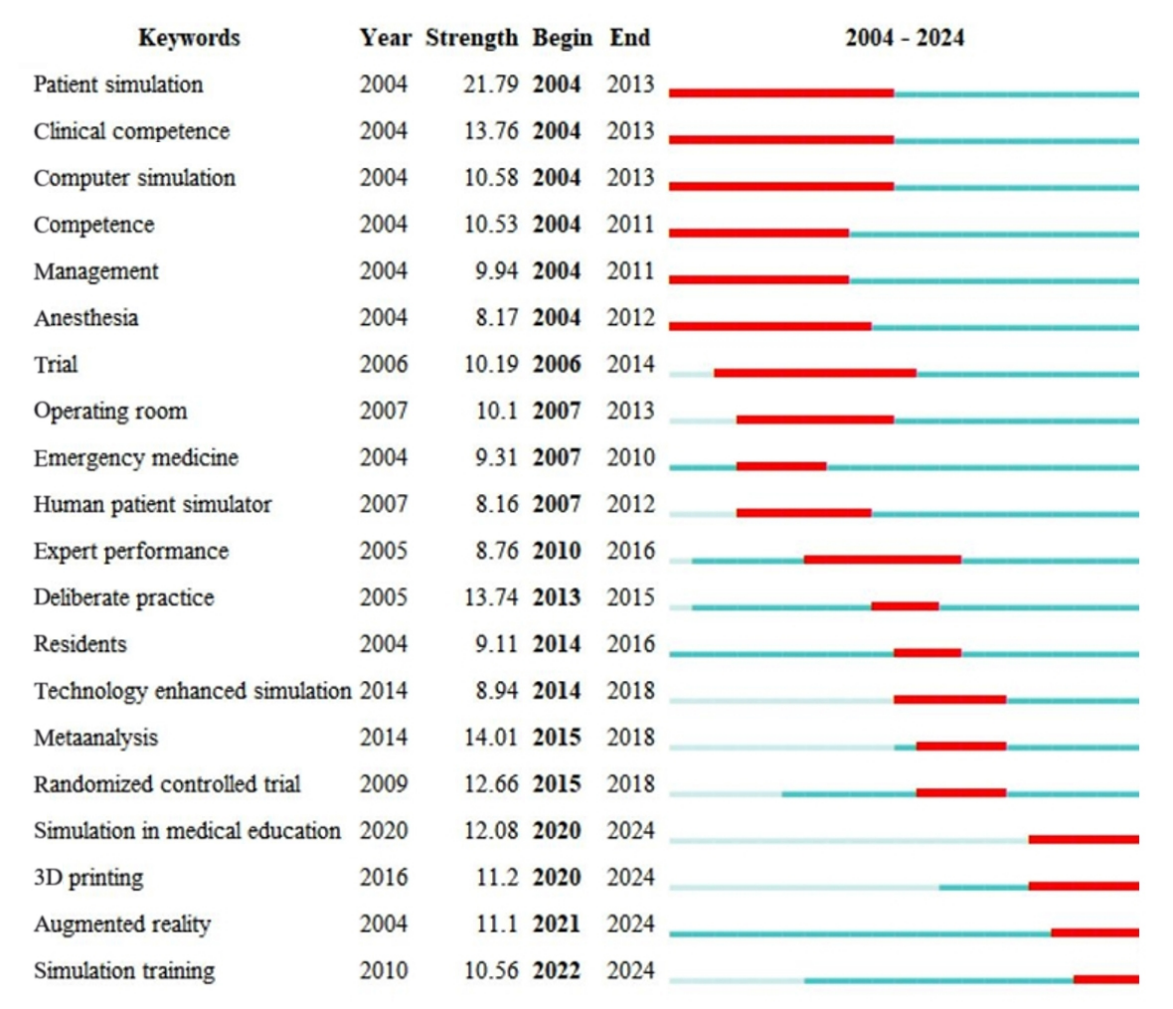
Keyword burst graph (sorted by the beginning year of the burst). The blue bars denote the reference has been published; the red bars denote citation burstiness.

The keyword burst visualization analysis identified a total of 20 keywords in the field of simulation teaching in medical education from 2004 to 2024, along with their emergence intensity and start-end years. The relevant literature on keyword emergence is shown in Figure 6.

It can be observed that the keywords with high emergence intensity include “patient simulation,” “randomized controlled trial,” “clinical competence,” and “deliberate practice.” Meanwhile, the research area began to focus on “patient simulation” as early as 2004, which, along with “clinical competence” and “computer simulation,” became one of the keywords with the longest duration of emergence. In recent years, researchers have increasingly focused on themes such as “trial” and “expert performance.” The keywords that are still emerging represent the current research frontiers and trends, which include “simulation in medical education,” “3D printing,” “augmented reality,” and “simulation training.” The emergence of “3D printing” reflects the growing interest in using patient-specific anatomical models for surgical planning and training, offering a more personalized and immersive learning experience. Similarly, “augmented reality” signifies the integration of advanced technologies to create interactive and realistic training environments, enhancing the acquisition of clinical skills. These emerging trends highlight the transformative potential of technology in medical education, paving the way for more innovative and effective teaching methodologies.

## Discussion

### Principal Findings

The results of our bibliometric analysis provide a comprehensive overview of the evolution, collaboration patterns, and thematic focus of simulation-based education research in the medical field. Key trends include a steady increase in publications from 2004 to 2024, particularly a surge after 2009, indicating a growing recognition of simulation’s importance in medical education. By 2024, the publication count had peaked at 850, highlighting a transition of simulation from a novel approach to a staple in medical education. In addition, the United States emerged as the leading contributor with 3083 articles, reflecting substantial investment in education and research. Harvard University is a central hub for simulation-based medical education, despite a fragmented institutional landscape. Prominent authors like Lars Konge, Adam Dubrowski, and William C McGaghie drive the field, though the low density of collaborative networks suggests room for enhanced inter-institutional teamwork. Keyword analysis underscores the focus on competency-based education and practical skill acquisition, with emerging technologies like 3D printing and AR shaping future directions.

### Comparison to Literature

Our findings are consistent with existing literature [16,17], which also highlights the increasing role of simulation in medical education over the past two decades. Previous studies have documented the rise in publications and the central role of the United States and key institutions like Harvard in advancing this field. However, our analysis provides a more granular look at the collaborative networks and thematic focuses, revealing a fragmented institutional landscape and the emergence of cutting-edge technologies that are less emphasized in earlier reviews.

### Implications of Findings

The implications of these findings are multifaceted. The robust growth in simulation-based medical education research indicates a broad acceptance of its efficacy in improving medical training. The strong international collaboration suggests that best practices and innovative methodologies are being shared globally, potentially standardizing and enhancing simulation protocols. The emergence of new technologies like 3D printing and AR points to a future where simulation-based education will be more immersive and technologically advanced [18-20], which could significantly enhance learning outcomes and patient care. The integration of 3D printing and AR into simulation-based training can significantly improve clinical outcomes. 3D-printed anatomical models enable patient-specific simulations, allowing surgeons to practice complex procedures before operating on real patients, thus enhancing precision and reducing errors [21]. Similarly, AR creates immersive training environments, providing real-time feedback and interactive learning to enhance clinical skill acquisition [22]. However, challenges such as the high cost of equipment and the need for specialized training for educators and learners may limit their widespread adoption. Future research should explore cost-effective solutions to overcome these barriers and ensure broader access to these technologies in medical institutions.

The emergence of “cardiopulmonary resuscitation” as a major research hotspot reflects its critical importance in medical education and clinical practice. Cardiopulmonary resuscitation is a high-stakes procedure where errors can have severe consequences, making it an ideal candidate for simulation-based training [23]. Simulation allows learners to practice cardiopulmonary resuscitation in a controlled environment, receive immediate feedback, and refine their skills through repetitive practice [24]. This not only enhances individual competence but also improves team dynamics and communication during real-life emergencies.

Similarly, the focus on “surgical education” underscores the need for advanced training methods to prepare surgeons for complex procedures. Simulation-based training in surgical education has been shown to improve technical skills, reduce operative time, and enhance patient safety [25]. These findings highlight the transformative potential of simulation in addressing critical gaps in medical education and improving clinical outcomes.

### Limitations

While the bibliometric analysis provides valuable insights, it has several limitations. First, the data might not capture all relevant publications, particularly those in non-English languages or those in less accessible databases, which could introduce selection bias. Second, the analysis relies on citation metrics, which may not fully reflect the quality or practical impact of the research. For instance, highly cited articles may not always represent the most impactful studies in terms of educational outcomes. Third, the low density of collaborative networks suggests that our findings might underrepresent the potential for interinstitutional synergy and innovation. Finally, a limitation of this study is the reliance on a single database (WoSCC), which may not capture all relevant publications. Future studies could expand the search to include additional databases such as PubMed and Scopus to enhance the robustness of the findings.

### Suggestions

To address the identified limitations and enhance the impact of simulation-based education research, we suggest the following:

1. Increasing efforts to include diverse and international publications in future analyses.
2. Encouraging more interinstitutional collaborations to create a more cohesive research landscape.
3. Fostering larger, integrated research teams to deepen the scope of studies and drive innovation.
4. Embracing and further investigating emerging technologies to stay at the forefront of educational advancements.

### Conclusions

In conclusion, the bibliometric analysis of simulation in medical education research reveals a dynamic field characterized by rapid growth, strong international collaboration, and evolving thematic focuses. The increasing trend in publications, significant contributions from leading countries and institutions, and the integration of new technologies underscore the impactful nature of this research area. Moving forward, enhancing collaboration among institutions and expanding the core author network will be crucial. Future research should focus on integrating emerging technologies, such as 3D printing and AR, into medical education. For instance, studies could explore how 3D-printed anatomical models can enhance surgical training by providing realistic, patient-specific simulations.

## Abbreviations

AR: augmented reality
WoSCC: Web of Science Core Collection

## Data Availability

All datasets generated for this study were included in the manuscript.
